# Structural analogues in herbal medicine ginseng hit a shared target to achieve cumulative bioactivity

**DOI:** 10.1038/s42003-021-02084-3

**Published:** 2021-05-10

**Authors:** Wei Zhang, Wei-Wei Tao, Jing Zhou, Cheng-Ying Wu, Fang Long, Hong Shen, He Zhu, Qian Mao, Jun Xu, Song-Lin Li, Qi-Nan Wu

**Affiliations:** 1 Department of Pharmaceutical Analysis, Affiliated Hospital of Integrated Traditional Chinese and Western Medicine, Nanjing University of Chinese Medicine, Nanjing, 210028 People’s Republic of China; 2 College of Pharmacy, Nanjing University of Chinese Medicine, Nanjing, 210023 People’s Republic of China; 3 School of Integrated Chinese and Western Medicine, Nanjing University of Chinese Medicine, 210023 Nanjing, People’s Republic of China; 4 Department of Metabolomics, Jiangsu Province Academy of Traditional Chinese Medicine, Nanjing, 210028 People’s Republic of China; 5 School of Chinese Medicine, Hong Kong Baptist University, Hong Kong, People’s Republic of China; 6 Jiangsu Collaborative Innovation Center of Chinese Medicinal Resources Industrialization, Nanjing University of Chinese Medicine, Nanjing, 210023 People’s Republic of China

**Keywords:** Mechanism of action, Natural products

## Abstract

By a pilot trial on investigating immunomodulatory activity and target of ginsenosides, the major bioactive components of ginseng, here we report that structural analogues in herbal medicines hit a shared target to achieve cumulative bioactivity. A ginsenoside analogues combination with definite immunomodulatory activity in vivo was designed by integrating pharmacodynamics, serum pharmacochemistry and pharmacokinetics approaches. The cumulative bioactivity of the ginsenoside analogues was validated on LPS/ATP-induced RAW264.7 macrophages. The potentially shared target NLRP3 involved in this immunomodulatory activity was predicted by systems pharmacology. The steady binding affinity between each ginsenoside and NLRP3 was defined by molecular docking and bio-layer interferometry assay. The activation of NLRP3 inflammasomes in LPS/ATP-induced RAW264.7 was significantly suppressed by the combination, but not by any individual, and the overexpression of NLRP3 counteracted the immunomodulatory activity of the combination. All these results demonstrate that the ginsenoside analogues jointly hit NLRP3 to achieve cumulative immunomodulatory activity.

## Abstract (web summary)

Zhang et al. design ginsenoside structural analogues and demonstrate that their combination shows more potent immunomodulatory activities than individual ginsenosides used alone at the same dosages. They predict that these analogues act on the joint target NLRP3 and consequently suggest that structural analogues hit a shared target to achieve cumulative bioactivity.

## Introduction

Herbal medicines, also known as botanical medicines or phytomedicines, are mainly plant-derived materials or preparations with human health benefits^1^. From ancient to modern times, herbal medicines have contributed to human survival by disease prophylaxis and therapy^2^. Nowadays, the clinical practice of herbal medicines not only dominates traditional medicine systems (e.g. traditional Chinese medicine, Ayurveda, Islamic medicine) but also contributes to Western mainstream medicine as complementary and alternative remedies^3–5^.

Complexity of components is the leading feature of herbal medicines differing from chemical drugs^6^. To date, tens of thousands of compounds have been found in herbal medicines, and even hundreds in a single herb^7,8^. The chemical structures of these components are highly diverse, which are classified into various types, such as steroids, terpenes, flavonoids, etc., generally based on their characteristic carbon frameworks. In addition to the chemical-type diversity, a single type always comprises large number of structural analogues that have the same or similar structural scaffolds with different side chains or substituents^9^.

In the past century, researchers have endeavoured to understand the therapeutic mechanisms of herbal medicines^10^. Under the reductionism principle of “one drug-one target-one disease”, herbal components were isolated and individually evaluated by high-throughput screening using disease-related target(s)^11^. Although several compounds from herbal medicines have indeed been singled out as significantly bioactive (artemisinin is one example)^12^, the hit rates are very low. The overwhelming majority of these single chemicals disappointingly show much weaker bioactivities than the herbal medicines from which the chemicals are isolated^13–15^. Recently, “multiple components hitting multiple targets” has been increasingly explored and experimentally verified as the therapeutic rationale of herbal medicines^16^. For example, one study revealed that the combination of three components in a traditional Chinese medicines called Realgar-*Indigo naturalis* formula synergistically treats acute promyelocytic leukaemia (APL) by hitting different targets involved in the induction of APL cell differentiation^17^. However, these studies focused on chemicals belonging to different structural types; how structural analogues in herbal medicines work together remains unexplained so far.

Based on receptor theory that the interaction between ligands (drugs) and receptors (targets) are structurally selective^18^, here we hypothesize that structural analogues in herbal medicines hit a shared target to achieve cumulative bioactivity. To test the hypothesis, the immunomodulatory activity of ginsenosides, the major active structural analogues of commonly used medicinal herb ginseng^19^, and the action targets were examined as a pilot study. First, pharmacodynamics, serum pharmacochemistry and pharmacokinetics were integrated to qualitatively and quantitatively design a ginsenoside analogues’ combination that exerts definite immunomodulatory activity on immunocompromised mice. Second, lipopolysaccharide-adenosine triphosphate (LPS/ATP)-induced RAW264.7 macrophages were used to compare the immunomodulatory activities between the combination and individual ginsenosides at the same dosages. Third, the potentially shared target of the ginsenoside analogues involved in the immunomodulatory activity of the combination were predicted by systems pharmacology. Fourth, the binding affinity between each ginsenoside and the target was evaluated by molecular docking and bio-layer interferometry assay. Finally, in LPS/ATP-induced RAW264.7 macrophages, effects of the combination and individual ginsenosides on the target were investigated and compared, and the mediator role of the target in the immunomodulatory activity of the combination was further examined.

## Results

### Designing the ginsenoside analogues’ combination with definite immunomodulatory activity in vivo by pharmacodynamics, serum pharmacochemistry and pharmacokinetics

**Fig. 1:**
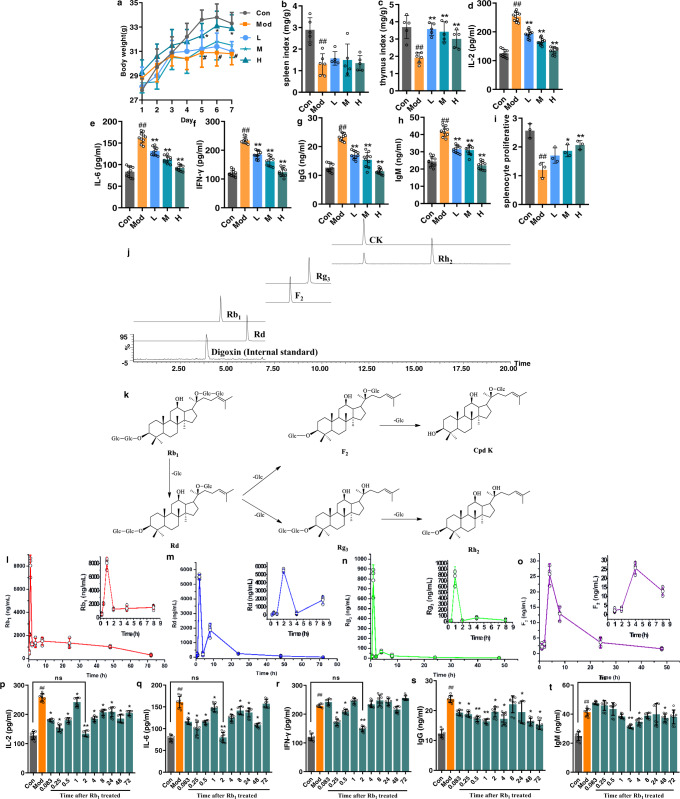
Pharmacochemical, pharmacokinetic and pharmacodynamic analyses determined the analogues’ combination as ginsenoside Rb_1_ (1247.32 ng/mL, 1.12 µM), Rd (5510.30 ng/mL, 5.82 µM), Rg_3_ (16.25 ng/mL, 20.72 nM) and F_2_ (3.07 ng/mL, 3.91 nM). **a** Body weight (*n* = 10), **b** spleen index (*n* = 5), **c** thymus index (*n* = 5), **d** IL-2 (*n* = 10), **e** IL-6 (*n* = 10), **f** IFN-γ (*n* = 10), **g** IgG (*n* = 10), **h** IgM (*n* = 10) and **i** splenocyte proliferative (*n* = 3). **j** HPLC-TQ-MS/MS chromatograms (negative ion mode) of ginsenoside Rb_1_ and its metabolites in serum. **k** Proposed in vivo metabolic pathway of ginsenoside Rb_1_. **l**–**o** Concentration–time curves of ginsenoside Rb_1_ and its metabolites (Rd, Rg_3_ and F_2_) after the oral administration of ginsenoside Rb_1_ (160 mg/kg) on the 7th day (*n* = 3). **p**–**t** Dynamic pharmacodynamic evaluation after the oral administration of ginsenoside Rb_1_ (160 mg/kg) on the 7th day (*n* = 8). Data are expressed as mean ± SD. Compared with Con, ^#^*P* < 0.05, ^##^*P* < 0.0^1^; compared with Mod, ^*^P < 0.05, ^**^P < 0.01, ^***^P < 0.001; ns represents no significant difference. Con: control group; Mod: model group; L: Rb_1_-treated group with low dose (40 mg/kg); M: Rb_1_-treated group with middle dose (80 mg/kg); H: Rb_1_-treated group with high dose (160 mg/kg).

In order to acquire a combination that has definite bioactivity, the immunomodulatory activity in vivo of orally administrated ginsenoside Rb_1_ in different dosages was first evaluated on cyclophosphamide (CP)-induced immunocompromised mice, and then the serum profile of Rb_1_ and its metabolites at the time point with optimal bioactivity was determined. On the 7th day, the model mice appeared exhausted, sluggish, asthmatic and somnolent, and showed remarkable reductions in body weight, spleen and thymus indexes compared to the control mice. Serum expressions of cytokines IL-2, IL-6, IFN-γ and antibodies IgG, IgM were greatly elevated, while proliferation of splenic lymphocytes was significantly inhibited in the model group compared to the control group (Fig. 1a–i). After the intervention by Rb_1_, CP-induced immunocompromised phenotypes were ameliorated to varying degrees. Compared to mice in the model group, the mice in the treatment groups, especially in the high-dose group, showed improvement in mental and physical states, body weight and organ indexes (Fig. 1a–c). Rb_1_ greatly decreased the production of IL-2, IL-6, IFN-γ and the levels of IgG and IgM in a dose-dependent manner (Fig. 1d–h). Moreover, the treatments also reversed the proliferative responses of splenic lymphocytes (Fig. 1i). The results collectively suggested that the high dose of Rb_1_ exerts the strongest immunomodulatory activity in vivo among the dosages tested.

The mouse serum in the high-dose group was then characterized by UPLC-QTOF-MS/MS to seek the absorbed Rb_1_ and its metabolites. A total of five metabolites together with Rb_1_ were identified from the serum (Fig. 1j), and the metabolites were structurally elucidated as ginsenosides Rd, Rg_3_, F_2_, Rh_2_ and CK by comparing the mass spectra and retention times with those of reference compounds (Supplementary Fig. 1 and Supplementary Table 1). These metabolites possess the same dammarane skeleton as Rb_1_ and are structural analogues generated via the stepwise cleavage of glucopyranosyl moieties from the backbone of Rb_1_^20^ (Fig. 1k).

The concentration–time curves of the ginsenoside analogues were established during 72 h after the final administration of Rb_1_ on the 7th day (Fig. 1l–o). Meanwhile, the expressions of cytokines (IL-2, IL-6, IFN-γ) and antibodies (IgG, IgM) were dynamically examined (Fig. 1p–t). The results indicated that at the 2^nd^ h, the cytokine and antibody expressions were most significantly inhibited (no significant differences compared with the control group). The serum concentrations of analogues at this time point, i.e. Rb_1_ (1247.32 ng/mL, 1.12 µM), Rd (5510.30 ng/mL, 5.82 µM), Rg_3_ (16.25 ng/mL, 20.72 nM) and F_2_ (3.07 ng/mL, 3.91 nM), were thus determined as the dosages to be used in the subsequent in vitro test.

### Ginsenoside analogues’ combination achieved stronger immunomodulatory activity in vitro than individual ginsenosides at the same dosages

**Fig. 2:**
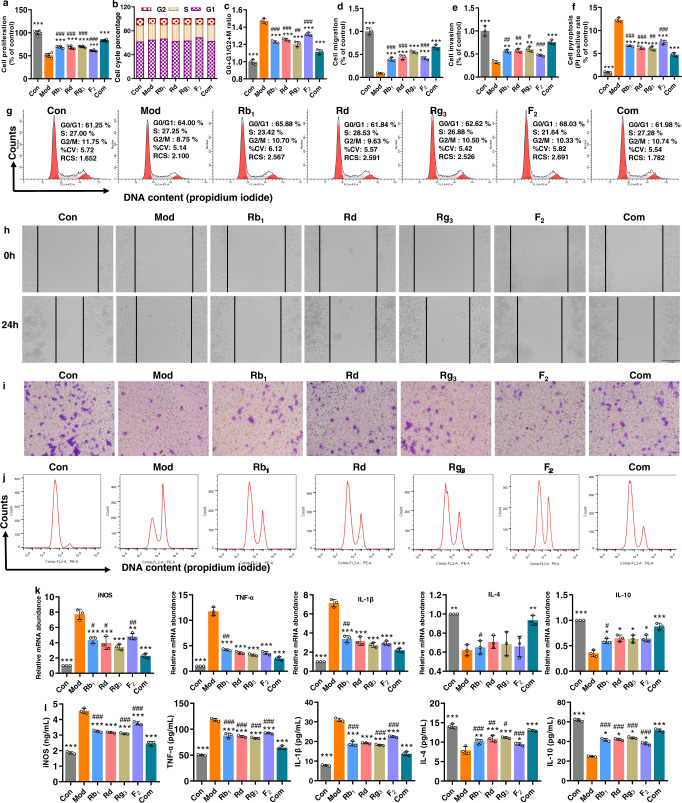
Ginsenoside analogues’ combination achieved stronger immunomodulatory activity in vitro than individual ginsenosides alone at the same dosages. **a** Cell proliferation (*n* = 6), **b**, **c**, **g** cell cycle, **d**, **h** cell migration, **e**, **i** cell invasion, **f**, **j** cell pyroptosis, **k** qRT-PCR assay for mRNA expressions and **l** ELISA for levels of pro-inflammatory (iNOS, IL-1β and TNF-α) and anti-inflammatory (IL-4 and IL-10) cytokines (**b**–**l**, *n* = 3). Data are expressed as mean ± SD. Compared with Mod, ^*^P < 0.05, ^**^P < 0.01, ^***^P < 0.001; compared with Com, ^#^*P* < 0.05, ^##^*P* < 0.01, ^###^*P* < 0.001.

LPS/ATP-induced RAW264.7 macrophages were used to investigate and compare the immunomodulatory activity in vitro between the combination and individual ginsenosides. MTT assay showed that LPS/ATP significantly inhibited the proliferation of RAW264.7 macrophages; the inhibition, however, was reversed by the intervention of ginsenosides, particularly the combination, suggesting that the ginsenoside treatments especially the combination had stimulating rather than toxic effects on the viability of RAW264.7 macrophages (Fig. 2a). DNA content analysis by propidium iodide (PI) staining revealed an increase in the fraction of cycling cells in LPS/ATP-treated macrophages from 61.25% to 64.00% in G0/G1 phase and a reduction from 11.75% to 8.75% of cells in G2/M phase, and the treatment by combination reversed them to 61.98% and 10.74%, respectively (Fig. 2b, c, g). Wound healing assay illustrated that each analogue promoted macrophage migration to the denuded zone of the scratched cell monolayer after 24 h treatment, and the combination reinforced the migration (Fig. 2d, h). Similarly, the cell invasion assay showed that the treatment with individuals significantly induced cell transfer, and the movement was further accelerated by treatment with the combination (Fig. 2e, i). PI staining assay indicated that the treatment with each analogue reduced the rate of cell pyroptosis, which however showed a further reduction in the combination group (Fig. 2f, j). qRT-PCR assay indicated that, compared to the control group, the LPS/ATP stimulation significantly increased mRNA expressions of iNOS, IL-1β and TNF-α, while it depleted those of IL-4 and IL-10 in the model group. The pre-inflammatory responses were reversed by the treatments of both combined and individual analogues, but the combination resulted in more notable improvement (Fig. 2k). The tendency for expression levels of the cytokines to vary was further confirmed by enzyme-linked immunosorbent assay (ELISA) (Fig. 2l). Taken together, the results suggest that the ginsenoside analogues’ combination exerted stronger immunomodulatory activity on RAW264.7 macrophages than each individual ginsenoside analogue at the same dosages.

### Predicting NLRP3 as a shared key target in mediating the immunomodulatory activity of ginsenoside analogues by systems pharmacology

**Fig. 3:**
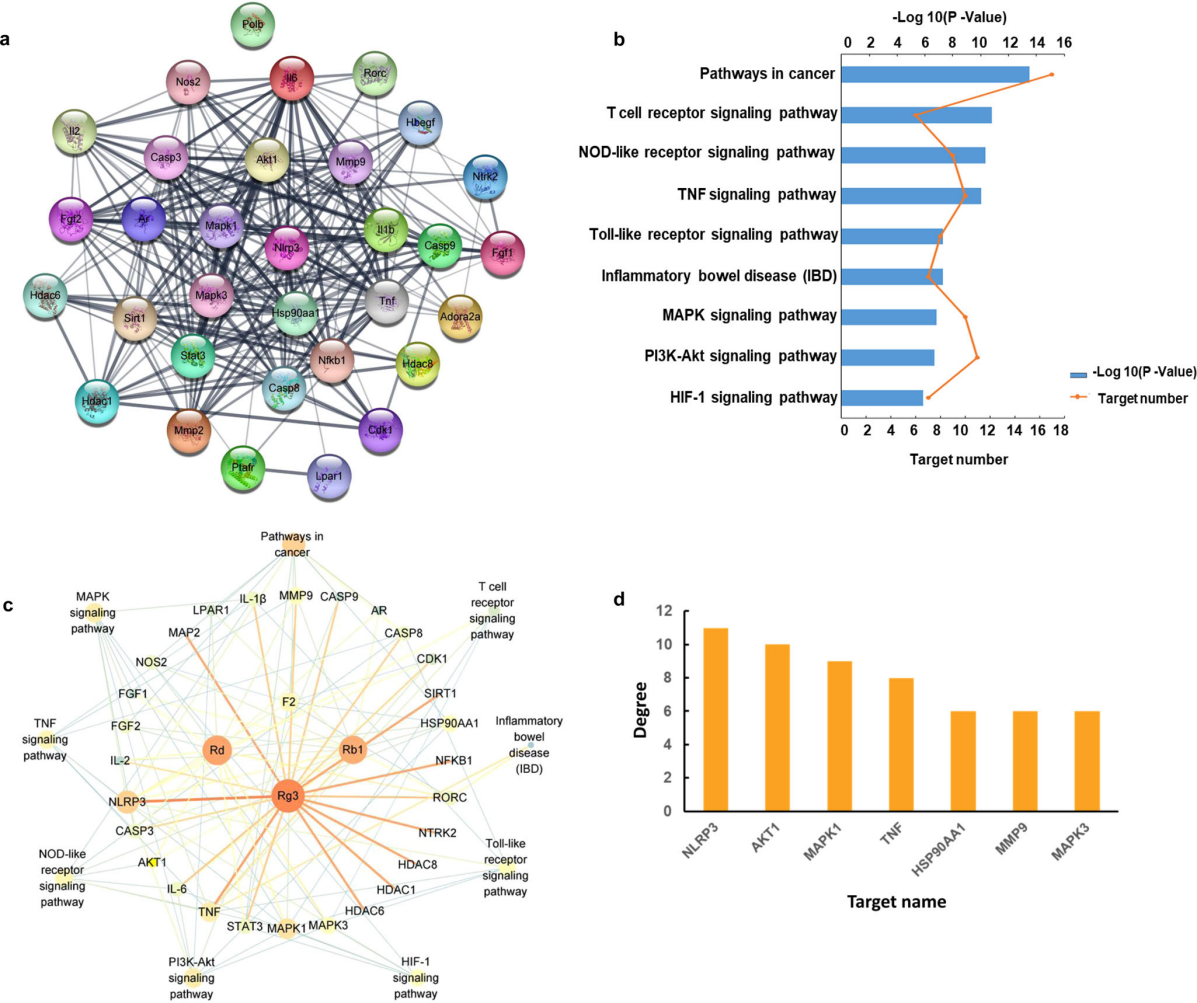
Systems pharmacology predicted NLRP3 as a key shared target in mediating the immunomodulatory activity of ginsenoside analogues. **a** PPI network analysis, **b** KEGG analysis, **c** compounds-targets-pathways network and **d** centrality algorithm analysis of the network.

The “protein–protein interaction (PPI) network” embedded in String revealed interactions between 32 immunomodulatory targets related to the treatment (Fig. 3a), 29 of which (*P* values ≥ 0.8) (Supplementary Table 2) were further investigated by Kyoto Encyclopedia of Genes and Genomes (KEGG) database to explore the relevant pathways. The 29 targets were found to involve 9 signalling pathways, including T cell receptor signalling, NOD-like receptor signalling, MAPK signalling, PI3K-Akt signalling, HIF-1 signalling, etc. (Fig. 3b). A compounds-targets-pathways network was then constructed and visualized with 42 nodes (4 ginsenosides, 29 targets and 9 pathways) and 1640 paths (Fig. 3c). The topological features of this network were calculated with the Network Analyzer plugin (Supplementary Table 3), which consisted of an entire portion of the interaction between the immunomodulatory targets, with an average number of direct neighbours of 5.659. The centrality algorithm analyses (degree coupled with closeness and betweenness) of the network screened out 7 key targets with degree value greater than the degree mean (Supplementary Table 4). Of these, NLRP3 ranked at the top among the three centrality algorithms (Fig. 3d), suggesting that the crucial signalling pathway involved in the ginsenoside treatment was associated with NLRP3. Previous studies have shown that uncontrolled activation of NLRP3 inflammasomes is one of the major triggers for a variety of autoimmune diseases and metabolic disorders^21^. Given this significance, NLRP3 was investigated to determine if the ginsenosides jointly target this molecule to overcome immunodeficiency.

### Evaluating the binding affinity of the ginsenoside analogues to NLRP3 by molecular docking and bio-layer interferometry

**Fig. 4:**
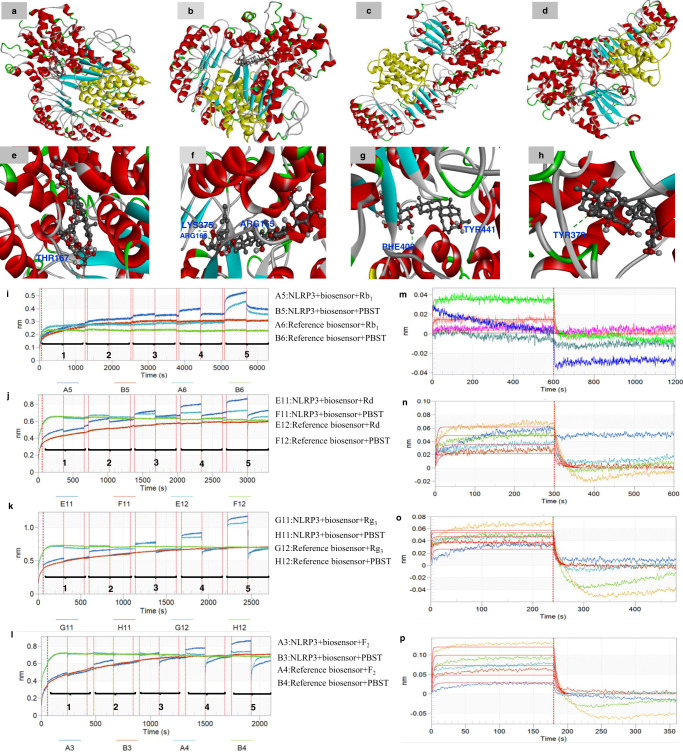
Binding affinity of the ginsenoside analogues to NLRP3 was evaluated by molecular docking and bio-layer interferometry. Molecular docking predicted the binding sites of ginsenoside analogues: **a**, **e** Rb_1_, **b**, **f** Rd, **c**, **g** Rg_3_, **d**, **h** F_2_ to NLRP3. The protein structure is demonstrated in ribbon format while ligand is represented in ball and stick format. Note: Red areas mean oxygen atom, grey areas mean carbon atom, blue areas mean nitrogen atom, white areas mean hydrogen atom and green areas mean other. The green dotted lines represent hydrogen bonds. Bio-layer interferometry validated the binding affinity of the ginsenoside analogues Rb_1_, Rd, Rg_3_, F_2_ to NLRP3. Typical kinetic characterization of NLRP3 to various concentrations of analogues by using BLI assay (*n* = 5). **i** Biosensors were exposed to various concentrations (marked 1–5) of Rb_1_ solution (1.44, 7.21, 36.1, 180.3, 901.7 μM) for association (600 s) and dissociation (600 s). **j** Biosensors were exposed to Rd solution (9.06, 18.1, 36.3, 72.6, 145.2 μM) for association (300 s) and dissociation (300 s). **k** Biosensors were exposed to Rg_3_ solution (7.18, 14.4, 28.7, 57.4, 114.8 μM) for association (240 s) and dissociation (240 s). **l** Biosensors were exposed to F_2_ solution (14.2, 28.3, 56.6, 113.2, 226.4 μM) for association (180 s) and dissociation (180 s). Association and dissociation steps are divided by the dotted line. Panels (**m**–**p**) represent Rb_1_, Rd, Rg_3_ and F_2_ in the association and dissociation steps in order to fit results globally with a 1:1 binding model and get the best values of *k*_on_, *k*_dis_ and *K*_D_.

The binding status between the analogues and NLRP3 was predicted by molecular docking (Fig. 4a–h and Supplementary Table 5). The results showed that hydrogen bonds are formed between Thr167 residues in NLRP3 and ginsenoside Rb_1_, and the conformation energy score of the NLRP3-Rb_1_ complex was −9.28 kcal/mol. Ginsenoside Rd formed three hydrogen bonds with residues Arg168, Arg165 and Lys375 in NLRP3, and it possessed an efficient binding affinity (−10.95 kcal/mol) with NLRP3. Similarly, Rg_3_ made two hydrogen bonds at the active sites of NLRP3 with its binding affinity of −12.33 kcal/mol. The hydrogen atom of the hydroxy group of ginsenoside Rg_3_ bonded with oxygen atom of Phe408 of NLRP3 (O-H-O-Phe408, 2.74 Å). Another bonding interaction occurred between the hydrogen atom of the hydroxyl radical of ginsenoside Rg_3_ and the oxygen atom of Tyr441 of NLRP3 (O-H-O-Tyr441, 2.65 Å). Ginsenoside F_2_ was adjacent to the hydrophilic residue Tyr379 in the active pocket of NLRP3, and F_2_ also had a steady binding affinity for NLRP3 with the docking score of −13.64 kcal/mol.

In order to more precisely validate that the ginsenosides directly target NLRP3, the equilibrium dissociation constant (*K*_D_) between the ginsenosides and NLRP3 were then determined by bio-layer interferometry. The protein immobilization exhibited good reproducibility with an average loading height at 2.54 nm, and the immobilization % CV of 3.69%, *n* = 8 (Supplementary Fig. 2). As shown in Fig. 4i–p and Supplementary Table 6, the interactions between NLRP3 and ginsenoside Rb_1_, Rd, Rg_3_ and F_2_ with the achieved *K*_D_ values of 9.96E-05, 5.01E-05, 9.61E-06 and 6.16E-05, respectively, were found. These data support the conclusion that all the ginsenoside analogues can bind to NLRP3 directly with strong affinity.

### The combination, but not individuals, significantly inhibits NLRP3 inflammasome activation in LPS/ATP-stimulated RAW264.7 macrophages

**Fig. 5:**
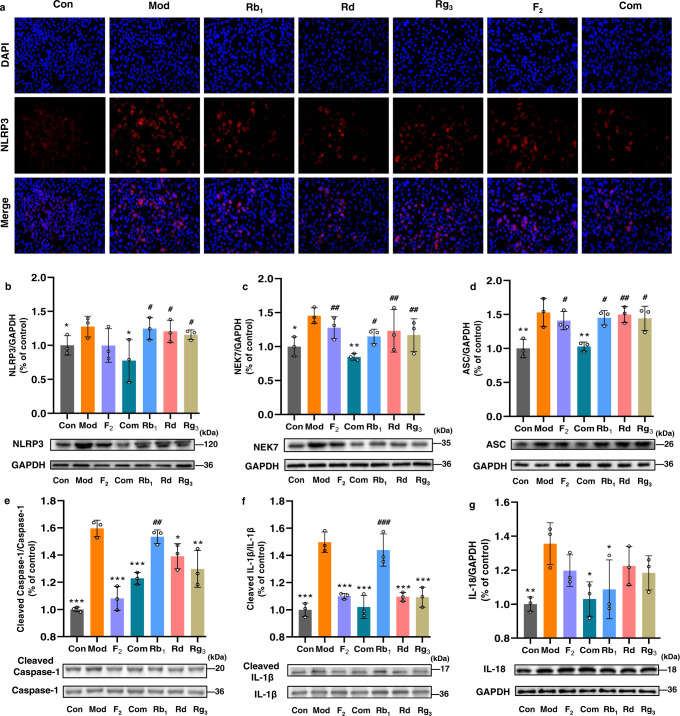
The combination, but not individuals, significantly inhibits NLRP3 inflammasome activation in LPS/ATP-stimulated RAW264.7 macrophages. Immunostaining assay for activated NLRP3 (**a**) and western blot assay for determining protein expressions: **b** NLRP3, **c** NEK7, **d** ASC, **e** cleaved caspase-1/caspase-1, **f** cleaved IL-1β /IL-1β, **g** IL-18 in cell lysates. GAPDH was used as the loading control. All data are expressed as mean ± SD (*n* = 3). Compared with Mod, ^*^P < 0.05, ^**^P < 0.01, ^***^P < 0.001; compared with Com, ^#^*P* < 0.05, ^##^*P* < 0.01, ^###^*P* < 0.001.

The impacts of the treatments on the activation of NLRP3 were then examined by immunofluorescence and western blot. Cells stimulated with LPS/ATP had a higher fluorescence intensity after NLRP3 labelling, which was however weakened by the treatments, particularly the one with the combination (Fig. 5a). The result was further validated by western blot, in which the expression of NLRP3 was significantly suppressed by the combination (Fig. 5b). Two proteins, namely NEK7 and ASC, are required for the assembly of NLRP3 inflammasomes^22,23^. We then examined the impacts of the combination and individual analogues on the expressions of NEK7 and ASC in LPS/ATP-stimulated RAW264.7 macrophages. The results showed that the expressions of the two proteins were significantly up-regulated by LPS/ATP; however, the overexpressions were greatly inhibited by the intervention of the combination. Interestingly, the reverse was not achieved in the treatments of any individual analogue at the same dosages (Fig. 5c, d). Since the assembly of the NLRP3 inflammasomes leads to caspase-1-dependent release of the pro-inflammatory cytokines IL-1β and IL-18^24^, we further examined and compared the effects of the combination and individuals on the NLRP3-caspase-1-IL-1β/IL-18 signalling pathway. It was found that pro-caspase-1/cleaved caspase-1 ratio, pro-IL-1β/cleaved IL-1β ratio and IL-18 expressions were all increased significantly in the model group compared with the control group. After the treatments some indicators were improved in the individual analogue-treated group, however, only the combination treatment improved all with significant differences(Fig. 5e–g). This evidence supports the conclusion that the treatment by the combination inhibited the activation of NLRP3 inflammasomes as well as the NLRP3-caspase-1-IL-1β/IL-18 signalling pathway in LPS/ATP-stimulated RAW264.7 macrophages, whereas the effects were not equivalently observed in the presence of individual analogues at the same dosages.

### Overexpression of NLRP3 counteracted the immunomodulatory activity of the combination in LPS/ATP-stimulated RAW264.7 macrophages

**Fig. 6:**
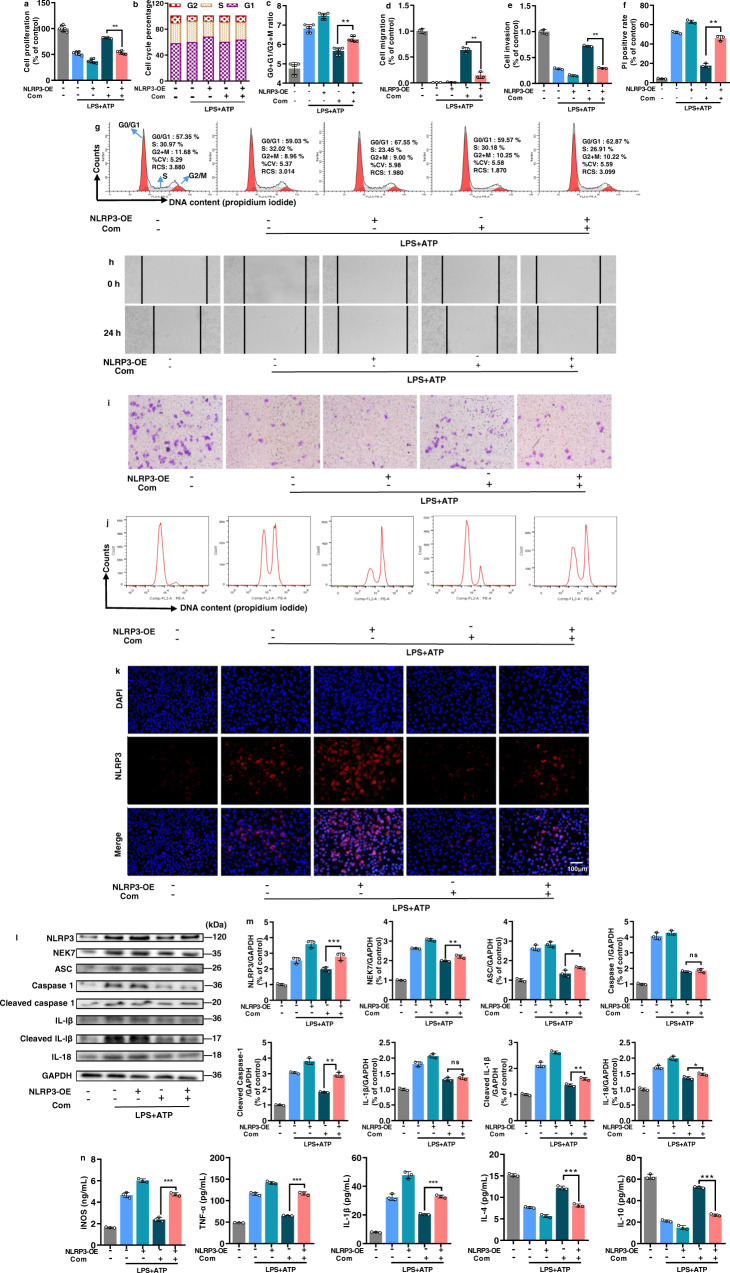
Overexpression of NLRP3 counteracted the immunomodulatory activity of the combination in LPS/ATP-stimulated RAW264.7 macrophages. **a** Cell proliferation (*n* = 6), **b**, **c**, **g** cell cycle (*n* = 6), **d**, **h** cell migration, **e**, **i** cell pyroptosis, **f**, **j** cell apoptosis, **k** immunostaining assay for activated NLRP3. **l**, **m**Western blot assay for determining protein expressions of NLRP3, NEK7, ASC, caspase-1, cleaved caspase-1, IL-1β, cleaved IL-1β and IL-18 in cells lysates (GAPDH was measured as the loading control). **n** ELISA for the levels of pro-inflammatory (iNOS, TNF-α and IL-1β) and anti-inflammatory (IL-4 and IL-10) cytokines in cell supernatants (**d**–**n**, *n* = 3). All data are expressed as mean ± SD. ^*^P < 0.05, ^**^P < 0.01; ns represents no significant difference.

In order to confirm that NLRP3 mediates the immunomodulatory activity of the combination, RAW264.7 macrophage was transfected with lentiviral vectors to induce the overexpression of NLRP3 (Supplementary Fig. 3). It was observed that the immunomodulatory activity of the combination on LPS/ATP-stimulated RAW264.7 macrophages was greatly counteracted in the NLRP3-overexpressed cells regarding cell proliferation (Fig. 6a), cell cycle (Fig. 6b, c, g), cell migration (Fig. 6d, h), cell invasion (Fig. 6e, i) and cell pyroptosis (Fig. 6f, j). In a similar way, the overexpression of NLRP3 also impeded the suppression of NLRP3 inflammasome signalling (NLRP3, NEK, ASC, cleaved caspase-1, cleaved IL-1β and IL-18) (Fig. 6k–m) and inflammatory responses (iNOS, IL-1β, TNF-α, IL-4 and IL-10) (Fig. 6n) by the combination intervention. Overall, the results demonstrated that NLRP3 is a crucial target by which the combination overcomes immunodeficiency in LPS/ATP-stimulated RAW264.7 macrophages.

## Discussion

The binding of drugs to disease-related targets, most of which are proteins such as enzymes, ion channels and receptors, occurs generally by intermolecular forces (ionic bonds, hydrogen bonds, Van der Waals forces, etc.)^25^. Therefore, chemical structure of the drug is a crucial factor to determine whether the binding happens or not, how strong the binding affinity is, as well as what pharmacological effects are triggered by the binding^26^. In this light, we have reasons to believe that, while herbal components of different chemical types are prone to act on different targets^17^, analogues can hit the same target due to their structural homogeneity. If so, the multiple hits on the target by multiple analogues could achieve cumulative, and thus significantly greater, bioactivity that might be not reached by any individual analogue at a certain concentration. Therefore, it is hypothesized that structural analogues in herbal medicines hit a shared target to achieve cumulative bioactivity. However, to the best of our knowledge, no proof-of-concept study has been conducted to test this hypothesis.

Ginseng, the root and rhizome of *Panax ginseng* C. A. Mey. (Araliaceae), is one of the most famed herbal medicines worldwide. In Oriental medicine, ginseng is widely regarded as a panacea, mainly due to its immunomodulatory properties^27^. Ginsenosides, a class of steroid-like saponins, have been experimentally demonstrated as the crucial components responsible for the immunomodulatory activity of ginseng^19^. Dammarane type is the major structural type of ginsenosides, from which abundant (more than 100) analogues that possess the same four-ring dammarane backbone decorated with different groups (glycosyls and hydroxyls) have been identified^28^. Ginsenosides always possess limited intestinal absorption after oral administration, because the hydrogen bonds, polar surface area and molecular flexibility of sugar moieties result in poor intestinal permeability^29^. Gut microbiota-catalysed deglycosylation, a main type of hydrolysis via the stepwise cleavage of glycosyl or glucuronosyl moieties from the backbone, is the deconjugation stage for most ginsenosides in the intestine^30^. The secondary ginsenosides and/or aglycones generated by this process normally possess better intestinal absorption and thereby better bioavailability^31^. Interestingly, a large portion of gut microbial metabolites of ginsenosides (especially secondary ginsenosides) also originally exist in ginseng. For example, several secondary ginsenosides, namely ginsenosides Rd, Rg_3_, F_2_ and Rh_2_, are not only found as major in vivo deglycosylated metabolites of ginsenoside Rb_1_^20,32^, a representative dammarane-type ginsenoside, but also naturally occur in ginseng as structural analogues to Rb_1_ with less glucopyranosyls^19^.

One of the major challenges to test the aforementioned hypothesis is how to rationally set the qualitative and quantitative composition of the analogues’ combination with therapeutic potential. Non-evidence-based selection is blind and cannot reveal actual situation. Here we proposed a resolution by integrating pharmacodynamics, serum pharmacochemistry and pharmacokinetics approaches. We evaluated the dynamic immunomodulatory activity in vivo of orally administrated ginsenoside Rb_1_ for 72 h; we qualitatively and quantitatively characterized Rb_1_ and its intestinal metabolites absorbed into the blood, and then constructed their concentration–time curves in the serum. Taken together, the results allowed us to capture the serum profile of these ginsenosides at the time point with the strongest immunomodulatory activity, and such a combination consisting of Rb_1_ and its metabolites (analogues) was assumed to significantly interact with immune-related target(s) in the circulation. Therefore, this analogues’ combination was then selected as a model to further explore if and how the analogues jointly hit one target to achieve cumulative immunomodulatory activity. As a result, the oral dosage of Rb_1_ was determined as 160 mg/kg as it showed the strongest immunomodulatory activity in vivo among the dosages tested. The serum pharmacochemical and pharmacokinetic analyses then indicated that Rb_1_ and its deglycosylated metabolites (Rd, Rg_3_, F_2_, Rh_2_ and CK) varied qualitatively and quantitatively during the 72 h after the oral administration. The dynamic pharmacodynamic evaluation at the same duration showed that the best improvements on immunocompromised phenotypes were achieved at the 2nd hour. At this time point, Rb_1_ and three metabolites, Rd, Rg_3_ and F_2_, were identified in the serum, whereas Rh_2_ and CK were not detected, probably because their serum concentration was lower than the detection limits of the assay. Therefore, ginsenosides Rb_1_, Rd, Rg_3_ and F_2_ at the serum concentrations as determined at the 2nd hour were selected as the combination investigated in this study.

Macrophage proliferation, migration and invasion are essential for immune response^33^, and LPS/ATP-induced macrophages secrete pre-inflammatory cytokines to propagate inflammation^34^. We observed that, in RAW264.7 macrophages, the combination treatment enhanced cell proliferation, migration and invasion, and inhibited LPS/ATP-stimulated over mRNA expressions of pre-inflammatory cytokines (iNOS, IL-1β, TNF-α), and increased mRNA expressions of anti-inflammatory cytokines (IL-4, IL-10) at highly significant levels. In individual-treated groups, the indicators were selectively improved but none were equivalent to the combination-treated group. In other words, the combination showed more potential in enhancing macrophage function than any individual analogue at the same dosage, which supports the conclusion that the combination achieved cumulative immunomodulatory activity in vitro.

Systems pharmacology^35^ was then used to predict the potential targets whereby the ginsenoside analogues exert the immunomodulatory activity. The calculation on topological features (degree, betweenness and closeness)^36^ of the established network of “drug-target-disease” suggested that NLRP3 is a crucial target in mediating the immunomodulatory activity of the ginsenosides. NLRP3 is an intracellular sensor that detects a broad range of microbial motifs, endogenous danger signals and environmental irritants, resulting in the formation and activation of the NLRP3 inflammasomes^37^. Assembly of the NLRP3 inflammasomes further activates caspase-1, which cleaves and maturates the pro-inflammatory cytokines IL-1β and IL-18, and then induces inflammatory, pyroptotic cell death^38^. NLRP3 inflammasomes is thus a vital player in innate immunity and inflammation, and its dysregulation has been implicated in a wide range of diseases, including Alzheimer’s disease, Prion diseases, type 2 diabetes and some infectious diseases^39,40^. Furthermore, suppressing the activation of NLRP3 inflammasomes has been investigated as a potential strategy to be used in treating diseases with ginsenosides such as Rb_1_ and Rd^41^. We thus further investigated if the analogues jointly target NLRP3 for the combination therapy.

Since the crystal structure of NLRP3 has not been reported, Cryo-EM structure of NLRP3 bound to NEK7 was used in molecular docking to predict the binding affinity and sites between the ginsenosides and NLRP3^42^. The results showed that the four ginsenosides can all bind to NLRP3 with an average docking score of −11.55 kcal/mol. More interestingly, although having hydroxyl groups on the same sites, the different ginsenosides can form different numbers (1–3) of hydrogen bonds with different amino acid residues of NLRP3. We assumed that specific steric effects generated by glucopyranosyls in the ginsenosides is the major reason for the difference^43^. The molecular docking prediction was further supported by *K*_D_ determined through bio-layer interferometry, in which the ginsenosides were demonstrated to bind to NLRP3 directly with strong affinity. These findings encouraged us to expect that, in the case of combination, the analogues jointly targeted NLRP3 to achieve the cumulative immunomodulatory activity.

We then determined whether the treatment with the combination suppressed the activation of NLRP3 inflammasomes. Adaptor protein ASC is crucial in the assembly of NLRP3 inflammasomes. Upon activation, the NLRP3 proteins oligomerize and recruit ASC, which then binds with caspase-1 to form the inflammasomes^22^. In addition, NEK7 is an NLRP3-binding protein that acts downstream of potassium efflux to regulate NLRP3 oligomerization and activation, and in the absence of NEK7, caspase-1 activation and IL-1β release are abrogated in response to signals that activate NLRP3^23,24^. Here we observed that, by the combination treatment, the overexpression of NLRP3, ASC, NEK7 and caspase-1 in LPS/ATP-induced RAW264.7 macrophages were significantly inhibited, and the production of cytokines IL-1β and IL-18 were accordingly decreased. The result constituted evidence that the treatment blocked the activation of NLRP3 inflammasomes as well as the NLRP3-caspase-1-IL-1β/IL-18 signalling pathway. However, the action was not achieved in any individual analogue-treated group. This result met our expectations, i.e. in contrast to dispersed and weak actions delivered by each analogue, the combined analogues cumulatively and significantly suppressed the shared target NLRP3. Next, the macrophage transfection experiment revealed that the overexpression of NLRP3 largely counteracted the amelioration of immunodeficiency by the combination in LPS/ATP-induced RAW264.7 macrophages, which further confirmed that the combination exerts the immunomodulatory activity by targeting NLRP3. However, how exactly the ginsenoside analogues jointly bind to NLRP3 as well as how the binding mediates the immunomodulatory activity still warrant further investigation.

In summary, by integrating pharmacology and molecular biology approaches, here we devised a pilot trial to demonstrate that structural analogues in herbal medicines hit a shared target to achieve cumulative bioactivity. In the current era, the structural diversity of chemical components in herbal medicines has been adequately explicated with the aid of advanced analytical techniques such as LC-MS^44^ and NMR^45^. Meanwhile, the pathogenesis of diseases has been revealed to always involve various factors—which means that there are multiple potential targets for treatment^46–48^. Under such a presupposition, the principle of “multiple components hitting multiple targets” is well accepted by the mainstream to understand how herbal medicines function. In particular, the theory is widely supported in recent years by systems biology, particularly network pharmacology and omics^49,50^. However, the findings highlighted in our study strongly suggest that “multiple analogues hitting a shared target” could additionally be involved in the pharmacological effects of herbal medicines. Therefore, this mechanism is complementary to “multiple components hitting multiple targets”; it enriches our understanding, expands the scientific interpretation of the rationale of herbal medicines and redirects research in the field of herbal medicine-based new drug discovery. In the past century, the hit rates of new drug candidates from herbal medicines are extremely low based on the single-compound test using classic reductionist methodology. Encouragingly, modern drug discovery is attaching increasing importance to identifying potential modulators for multiple targets from millions of natural compounds as a combined drug^51,52^. In the future, we may also consider analogues’ combination for hitting a shared target as a complementary strategy for developing new drugs from herbal medicines.

## Methods

### In vivo immunomodulatory activity evaluation

Animal facilities and protocols were approved by the Affiliated Hospital of Integrated Traditional Chinese and Western Medicine of Nanjing University of Chinese Medicine. All procedures were conducted in strict accordance with Guide for the Care and Use of Laboratory Animals of the National Institutes of Health (NIH Publication No. 80-23; revised in 1978). Fifty male ICR mice, 6–8 weeks of age, were purchased from Shanghai Laboratory Animal Research Center (Shanghai, China). Each animal was evaluated to be in good health, and then acclimated to the laboratory environment (12 h light/dark cycle, at 23–27 °C, and 30–60% relative humidity) for 1 week before experiments. Feed and potable water were provided ad libitum.

The mice were randomly divided into five groups (10 for each group): control group (Con), model group (Mod) and three Rb_1_-treated groups, namely the low-dose (L, 40 mg/kg), middle-dose (M, 80 mg/kg) and high-dose (H, 160 mg/kg) groups. Body weight of each mouse was recorded per day, and the drug dose was modified daily according to the body weight. Mice in the model and Rb_1_-treated groups were intraperitoneally injected with 100 mg/kg CP on the 4th day, while the control group was given the same volume of normal saline. The control and treated groups were intragastrically administered double distilled water and Rb_1_, respectively, for 7 consecutive days.

Mice were sacrificed by cervical dislocation on the 7th day; the thymus and spleen were harvested and weighed immediately. The immune organic indexes were calculated as organ weight/body weight (mg/g). Blood was taken from eyeballs of mice and immediately separated by centrifugation (3000*g*) at 4 °C for 10 min. The resulting serum was stored at −80 °C until use. The levels of cytokines interleukine-2 (IL-2), interleukine-6 (IL-6), interferon-γ (IFN-γ) and antibodies IgG, IgM in the serum were measured using ELISA kits according to the manufacturer’s protocols^53^ (Multiskan Ex, Lab Systems, Finland).

Lymphocyte proliferation was evaluated by MTT assay. Spleen was minced and passed through a steel mesh under aseptic conditions and kept in Hank’s balanced salt solution (HBSS, Sigma-Aldrich). Red blood cell lysis buffer (1:5 *v*/*v*) was added, after centrifugation (500*g* at 4 °C for 5 min) cells were washed in HBSS and re-suspended in RPMI 1640 medium with 0.05 mM 2-mercaptoethanol, 100 IU/mL penicillin, 100 µg/mL streptomycin and 10% fetal bovine serum (FBS). Cell viability was estimated using Trypan blue exclusion, and the concentration of viable lymphocytes was more than 95%. Then, 5.0 × 10^6^ cells/mL were seeded in a 96-well plate in the presence of LPSs (5 µg/mL) or medium, and kept at 37 °C with 5% CO_2_ for 48 h. Then, 50 µL MTT solution (2 mg/mL) was added 4 h before the end of incubation, and untransformed MTT was removed after centrifugation (1000*g* for 8 min). At last, 200 µL dimethyl sulfoxide (DMSO) working solution (192 µL DMSO with 8 µL 1 M HCl) was added to each well. The absorbance was evaluated in an ELISA reader at 570 nm with a 630 nm reference.

### Serum pharmacochemistry

After adding 4 mL of methanol, 500 μL serum was vortex-mixed for 5 min and then centrifuged at 12,000*g* for 10 min. Subsequently, the supernatant was transferred into another tube and the organic phase was evaporated to dryness at 40 °C under a stream of nitrogen. The residue was re-dissolved with 200 μL methanol and then filtered through a 0.2 μm PTFE syringe filter. An aliquot of 2 μL was injected into the UPLC-QTOF-MS/MS system for analysis.

Liquid chromatography was performed with a Waters Acquity ultra-performance liquid chromatography (UPLC) core system (Waters Corp., Milford MA, USA), equipped with a binary solvent delivery system, an auto-sampler and a photo-diode array (PDA) detector. The column was a Waters Acquity HSS T3 (2.1 mm × 100 mm, I.D., 1.8 μm). The mobile phases consisted of (A) 0.1% formic acid in water and (B) acetonitrile containing 0.1% formic acid. The UPLC elution condition was optimized as follows: 25% B (0–1 min), 25–40% B (1–4 min), 40% B (4–11 min), 40–95% B (11–11.1 min), 95% B (11–13 min), 95–25% B (13–13.1 min) and isocratic at 25% B (13.1–15 min). The flow rate was set at 0.4 mL/min. The column and auto-sampler temperatures were maintained at 35 and 10 °C, respectively.

Mass spectrometry was performed on a Waters Synapt G2S QTOF (Micro mass MS Technologies, Manchester, UK) equipped with electrospray ionization source operating in negative mode. The nebulization gas was set to be 1000 L/h at a temperature of 450 °C, and the cone gas was set at 40 L/h. The capillary voltage and cone voltage were set at 3500 and 45 V, respectively. The QTOF acquisition rate was 0.2 s, and the inter-scan delay was 0.02 s. Argon was employed as the collision gas at a pressure of 7.110 × 10^−3^ Pa. The mass spectrometer and UPLC system were controlled by MassLynx 4.1 software. Data were collected in centroid mode, and the MSE approach was used. The energies for collision-induced dissociation (CID) were set at 5 and 60 eV, respectively, for fragmentation. The fragmentation pathways of each component were deduced with the help of MassLynx^TM^ (v 4.1) software^54^.

All MS data were acquired using LockSpray to ensure mass accuracy and reproducibility. The molecular masses of the precursor ion and product ions were accurately determined with leucine enkephalin (*m/z* 554.2615) in negative mode at the concentration of 50 pg/μL; the infusion flow rate was 10 μL/min. Centroid data were acquired for each analogue from 100 to 1500 Da. Dynamic range enhancement was applied in the mass spectrometer (MS) experiment to ensure accurate mass measurement over a wide dynamic range.

### Pharmacokinetics

Retro-orbital blood samples were collected into heparinized tubes immediately before and at 0.083, 0.25, 0.5, 1, 2, 4, 8, 24, 48 and 72 h after last administration. Plasma samples were prepared by centrifuging the blood at 4000 rpm for 10 min at 4 °C and stored at −80 °C for further use. Then, 200 μL plasma samples and 40 μL Digoxin (5 μg/mL) were mixed with 800 μL of methanol. Each mixture was then vortex-extracted for 5 min, and centrifuged at 12,000 rpm for 10 min at 4 °C. The supernatant was transferred and evaporated to dryness at 45 °C in a rotary evaporator. The residue was dissolved with 200 μL methanol solution. After centrifugation again at 12,000 rpm for 10 min, the supernatant was collected for HPLC-TQ-MS analysis.

The HPLC analysis was performed on a Waters Alliance HPLC 2695 system (Waters Corp., MA, USA), equipped with a binary solvent delivery system and an auto-sampler. The chromatographic separation was achieved on an Agilent Poroshell 120 EC-C18 column (100 mm × 3.0 mm, I.D., 2.7 μm) with a Phenomenex C_18_ guard column. The mobile phase consisted of (A) 0.1% formic acid and (B) acetonitrile with 0.1% formic acid. The gradient elution was optimized as follow: 30–65% B (0–9 min), 65–95% B (9–11 min), 95% B (11–15 min), 95-30% B (15–16 min), 30% B (16–20 min), flowing at 0.4 mL/min. The column and auto-sample temperatures were maintained at 35 and 10 °C, respectively. The injection volume was 10 μL.

Mass spectrometry was performed on a Micromass Quattro-Micro^TM^ triple-quadrupole mass spectrometer (Water CO., Milford, MA, USA) with electrospray ionization (ESI) interface. All analytes were monitored under negative ionization mode and analysed by multiple reaction monitoring (MRM) mode. Argon was the collision gas. Other parameters of the mass spectrometer were set as follows: the capillary voltage was 3 kV, source temperature was 120 °C and desolvation gas was 400 L/h at 400 °C. Detailed conditions^55^ for MRM analysis for each analyte are summarized in Supplementary Table 7.

### Macrophage proliferation assay

Mouse RAW264.7 cells (Cell Bank of the Chinese Academy of Sciences, Shanghai, China) were cultured in Dulbecco’s Modified Eagle Medium (DMEM) (Gibco, Termo Fisher Scientific, USA) supplemented with 10% FBS, 100 U/mL penicillin, 100 μg/mL streptomycin and 2 mM L-glutamine at 37 °C in a humidified incubator (Forma 3111 CO_2_ incubator, Thermo Forma, USA) with 5% CO_2_.

Next, 5 × 10^4^ cells/well in 96-well plates were pre-treated with the ginsenosides (individuals or combination) for 2 h. Next, LPS (1 µg/mL, dissolved in DMEM) and DMEM were used as the model and control, respectively. After culturing for 24 h, the medium was discarded, the fresh medium containing 5 mM ATP was then added for another 30 min except the control. Then, 200 µL MTT (5 mg/mL) was added, after 4 h the supernatant was removed, and 150 µL DMSO was added to each well and incubated in the dark for 10 min. Cell absorbance was determined at 450 nm using a microplate reader^56^ (Thermo Molecular Devices Co., Union City, USA).

### Macrophage cycle analysis

For this analysis, 1 × 10^6^ cells/mL were cultured in 6-well plates for 24 h, then the ginsenosides with indicated concentrations (individuals or combination) were added for another 2 h. After that, cells were stimulated by 1 µg/mL LPS (dissolved in DMEM) for 24 h, and fresh medium containing 5 mM ATP was replaced, and incubation continued for 30 min. Then, 0.05% trypsin (without EDTA) was added for digestion, and cell precipitate was collected by centrifugation. Cells were washed once with PBS, fixed with 70% ethanol for 1 h at 4 °C, then washed twice with PBS, treated with 100 µg of RNase A for 30 min at 37 °C, washed once with PBS, and finally stained with 20 µg of PI (Beyotime, C1052) in PBS. Cell cycles were detected by FACS CantoII flow cytometry^57^ (Becton Dickinson, CA).

### Macrophage migration assay

The cells were spread in 6-well plates with 6 × 10^6^ cells per well in a 37 °C incubator for 24 h. After cells had grown to 90% confluency, a gap was made using a 10 µL pipette tip. After generating the wound^58^, the ginsenosides with indicated concentrations (individuals or combination) were added for another 2 h. After that, cells were stimulated by 1 µg/mL LPS (dissolved in DMEM) for 24 h, and fresh medium containing 5 mM ATP was replaced, and incubation continued for 30 min. Cells that had migrated to the wound were visualized using an inverted phase microscope (Olympus, Tokyo, Japan), and the scratch area was quantified via Image-Pro Plus (v 6.0) software.

### Macrophage invasion assay

For this assay, 5 × 10^4^ cells/well in serum-free DMEM were added to a 24-well inner Transwell chamber (8 µm, Corning Inc.), which was pre-coated with or without 300 µg/mL Matrigel (BD Biosciences) for 2 h. The outer chamber was filled with 10% FBS in DMEM medium containing the indicated concentration of ginsenosides (individuals or combination) as an inducer. Cells were stimulated by 1 µg/mL LPS (dissolved in DMEM) for 24 h, and 5 mM ATP was added for another 30 min. The migrated cells were fixed, stained with 0.1% crystal violet and counted (magnification, ×200) in five different areas under an inverted fluorescence microscope (Nikon Eclipse TS100‑F; Nikon).

### Macrophage pyroptosis assay

Cells were cultured, moulded and administered in the same manner as for the cell cycle assay. After discarding the medium, 0.05% trypsin (without EDTA) was added for digestion. Cell precipitates were collected, and washed twice with PBS for PI (Beyotime, C1052) staining, and the pyroptosis rate was detected by FACS CantoII flow cytometry^56^ (Becton Dickinson, CA).

### Macrophage transfection

The recombinant adenovirus vectors for NLRP3 and mock vector were provided by Genechem Co., Ltd. (Shanghai, China). All vectors were labelled with GFP, which served as a detection marker. Here, 1 × 10^6^ cells/well grown to 50–70% confluence were incubated in 6-well plates with serum-free medium for 18 h and were then transfected with NLRP3-overexpressed lentiviral vectors (virus titre is 2 × 10^8^ TU/mL, multiplicity of infection = 20). The transfection was conducted with Lipofectamine™ 3000 Reagent and P3000™ reagent (Thermo Fisher Scientific, Inc.) following the manufacturer’s instructions. Another 48 h later, cells were lysed and proteins were isolated. The efficiency of the transfection was performed by western blot.

### qRT-PCR

Total RNA from the RAW264.7 cells was extracted by using ice-cold TRIzol reagent (15596026, Thermo Fisher Scientific, NY, USA). A260/A280 and A260/A230 ratios were used to determine RNA quality and purity using NanoPhotometer (P300, Implen, Germany). PrimeScript™ II 1st Strand cDNA Synthesis Kit (6210 A, Takara, Japan) was used to synthesize the first strand of cDNA. The primer sequences (Supplementary Table 8) were designed by Primer 5.0 software and synthesized by Generay Biotech Co., Ltd (Shanghai, China). qRT-PCR was carried out using an Applied Biosystems ViiA™ 7 Real-Time PCR system (ViiA™ 7 Real-Time PCR System, Thermo Fisher Scientific, New York, USA). qRT-PCR reactions were performed as follows: holding at 50 °C for 2 min, pre-denaturation at 95 °C for 10 min, 40 cycles of 95 °C for 15 s and 60 °C for 1 min. The relative mRNA expression level of the target gene was calculated using 2^−ΔΔCt^ with β-actin as the reference gene^59^.

### ELISA

After centrifuging at 1000 rpm for 5 min, the RAW264.7 cell supernatants were collected, and the contents of IL-2, IL-6, IFN-γ, IgG, IgM, iNOS, IL-1β, TNF-α, IL-4 and IL-10 were detected using ELISA kits^60^.

### Western blot

Cell proteins were extracted using RIPA lysis buffer (Beijing Solarbio Science & Technology Co., Ltd.) with a protease inhibitor cocktail (Beyotime Institute of Biotechnology). First, equal amounts of proteins (50 µg) were subjected to 10% sodium dodecyl sulfate-polyacrylamide gel (SDS-PAGE) electrophoresis and then transferred onto polyvinylidene fluoride membranes. Next, 5% fat-free milk was used to block the membranes for 2 h, which were then incubated with primary antibodies: IL-1β (#12242, Cell Signalling Technology, CST), cleaved IL-1β (#52718, CST), IL-18 (ab71495, Abcam) 1:1000; caspase-1 (#24232, CST), cleaved caspase-1 (#89332, CST), NLRP3 (ab214185, Abcam), NEK7 (ab133514, Abcam) 1:500; ASC (#67824, CST) 1:200; and GAPDH (#5174, CST) 1:2000, overnight at 4 °C. Then the blots were incubated with secondary antibodies for 2 h. Finally, protein bands were visualized with an enhanced chemiluminescence (ECL) system (KeyGEN, Nanjing, China) and scanned with a chemiluminescence imaging system (Gel Catcher 2850, China). The grey values of bands were analysed with NIH ImageJ v1.52 software^61^.

### Immunofluorescence microscopy

Cells were seeded in LabTek-II chamber slides (Nunc) at 6 × 10^5^ cells/well and cultured for 24 h. The next day, ginsenosides with indicated concentrations (individuals or combination) were pre-treated for 2 h, then the cells were stimulated by 1 µg/mL LPS (dissolved in DMEM). After 24 h, the medium was replaced with fresh medium containing 5 mM ATP to continue the incubation for 30 min. After adding 0.3% of Triton-X100 and allowing it to permeate the cells for 20 min, 5% BSA was added to block the cells at room temperature for 1 h. NLRP3 (ab270449, Abcam, 1:50) antibody was then added and incubated overnight at 4 °C. After washing 3 times with PBS for 5 min each, Goat Anti-Rabbit IgG H&L (Alexa Fluor 594) antibody (ab150080, Abcam, 1:1000) was added and incubated at room temperature for 1 h^62^, and the nuclei were stained with 1 mg/mL of DAPI. An EVOS FL Cell Imaging System (Thermo Fisher Scientific, Rockford, USA) was used to acquire the immunofluorescent images. The fluorescence intensity of protein expression was measured by ImageJ v1.52 software.

### Systems pharmacology

The chemical structures of ginsenosides were converted to SDF and SMILES format through PubChem Compound (https://www.ncbi.nlm.nih.gov/pccompound/). The potential targets were then retrieved and validated based on the PharmMapper Server (http://lilab.ecust.edu.cn/pharmmapper/submit_file.php), Similarity Ensemble Approach (http://sea.bkslab.org/), STITCH (http://stitch.embl.de/) and Swiss Target Prediction (http://www.swisstargetprediction.ch/) databases. The immune-related genes were obtained from the Therapeutic Target Database (http://bidd.nus.edu.sg/group/cjttd/) with the selected species as *Mus musculus*. Taken together, these targets were integrated to remove duplicate items and were input into UniProt KB (http://www.uniprot.org/) to standardize their names and organisms. After that, the targets were fed into String11.0 (https://string-db.org/) to predict related protein–protein interactions (PPI) for acquiring target genes, and targets directly associated with immunity were screened according to P value ≥ 0.8. Degree centrality algorithm was adopted as the major algorithm, supplemented by the closeness centrality and the betweenness centrality algorithms, so as to select and evaluate the key immunity targets. Gene Ontology (GO) enrichment analysis and KEGG pathway enrichment analysis were performed using the functional annotation tool of Database for Annotation, Visualization and Integrated Discovery (DAVID) Bioinformatics Resources 6.8 (https://david.ncifcrf.gov/) and KEGG (http://www.kegg.jp/). Only terms with *P* value ≤ 0.05 were chosen.

Two networks, compound-target (C-T) and target-pathway (T-P) networks, were constructed and visualized by an open-source bioinformatics package, Cytoscape 3.7.2. In the whole network, compounds, targets and pathways were represented by nodes, and the relationships between them were represented by the edges. The topological properties of the network were analysed by the plugin Network Analyzer of Cytoscape^63^.

### Molecular docking

The Cryo-EM structure of NLRP3 bound to NEK7 (protein database, PDB code: 6NPY) was obtained from RCSB Protein Database Bank (http://www.rcsb.org). The 3D structures of ginsenosides were generated by Chem3D Ultra 8.0. The rotatable bonds of ligands were detected and assigned with AutoDock tools. The protein was prepared by repairing the missing and terminal residues of polypeptide chains, deleting water molecules, assigning atom types and adding hydrogen atoms. Molecular docking was performed on AutoDock v4.2.6. Docking calculations were performed by the Lamarckian genetic algorithm (LGA) with run times of 100. The auxiliary program Autogrid was used to generate the docking area, which was defined as a 40 × 40 × 40 3D grid centred on the ligands’ binding site with a 0.375 Å grid space. All the other miscellaneous parameters were set as default. Results differing by ≤2 Å in a positional root mean-square deviation (RMSD) were clustered together. Docking binding energy was calculated via Auto tool to explore binding interactions between the compounds and the target. The output from AutoDock v4.2.6 was rendered with Discovery Studio v4.5 to give graphic displays^64^.

### Bio-layer interferometry assay

The recombinant mouse NLRP3 protein (purity > 90%; concentration, 0.4 µg/µL; his-tagged) was diluted to 10 µg/mL with 20 mM Tris-HCl, 0.15 M NaCl (adjust pH to 8.0) buffer. Then, 200 μL/well protein buffer was added in the 96-well plate, and Ni-NTA biosensors were pre-wetted in the protein buffer for 10 min. The protein was first immobilized onto the biosensors under the optimized conditions: baseline1 300 s, loading 3600 s. When biosensors reached the maximum response, further kinetic experiments with small molecules were carried out.

Individual ginsenosides were dissolved in PBS containing 5% DMSO to get four stock solutions, and a series of five different concentrations were tested for binding to the immobilized protein. Then, 200 μL of each compound and kinetics buffer (PBS containing 5% DMSO) were transferred into a 96-black polypropylene plate. The assay temperature was 30 °C and the shake speed was set at 1000 rpm using the Fortebio Octet K2 System. Supplementary Table 9 showed the measurement parameters. Data were loaded into and processed by the Octet Data Analysis (v7.0) software^65^.

### Statistics and reproducibility

The statistical analyses were performed using GraphPad Prism 8.0.1 (GraphPad Software Inc., San Diego, CA, USA) software. All experiments were performed with three to ten samples per group and results derived from at least three independent measurements. All individual data points are plotted as dot plot or bar-dot plot throughout the manuscript. Statistical testing method, sample size, replicates of experiments, and the *P* value are indicated in figure legends. The Student’s *t*-test and one-way analysis of variance (ANOVA) were applied to compare the means of different groups. While Student’s *t*-test was used to demonstrate the means of two groups in comparison, ANOVA test was performed to compare the means of multiple groups. *P* values < 0.05 were accepted to indicate statistically significant differences.

### Reporting summary

Further information on research design is available in the Nature Research Reporting Summary linked to this article.

## Supplementary information

Supplementary information

Description of Additional Supplementary Files

Supplementary Data 1

Reporting summary

## Data Availability

The datasets generated during and/or analysed during the current study are available from the corresponding author on reasonable request. Source data underlying plots shown in main figures are provided in Supplementary Data.
