# Supplementary material for: Structural analogues in herbal medicine ginseng hit a shared target to achieve cumulative bioactivity
Source: Commun Biol. 2021 May 10;4:549. doi: 10.1038/s42003-021-02084-3 (PMC8110997; doi:10.1038/s42003-021-02084-3)
Supplement: Supplementary file 1 — Supplementary information [file 42003_2021_2084_MOESM1_ESM.pdf]

Supplementary Figures and Tables

Structural analogues in herbal medicine ginseng hit a shared target to achieve cumulative bioactivity

Wei Zhang, Wei-Wei Tao, Jing Zhou, Cheng-Ying Wu, Fang Long, Hong Shen, He Zhu, Qian Mao, Jun Xu[*], Song-Lin Li[**], Qi-Nan Wu[***]

Contents:

Supplementary Figures 1-3

Supplementary Tables 1-9

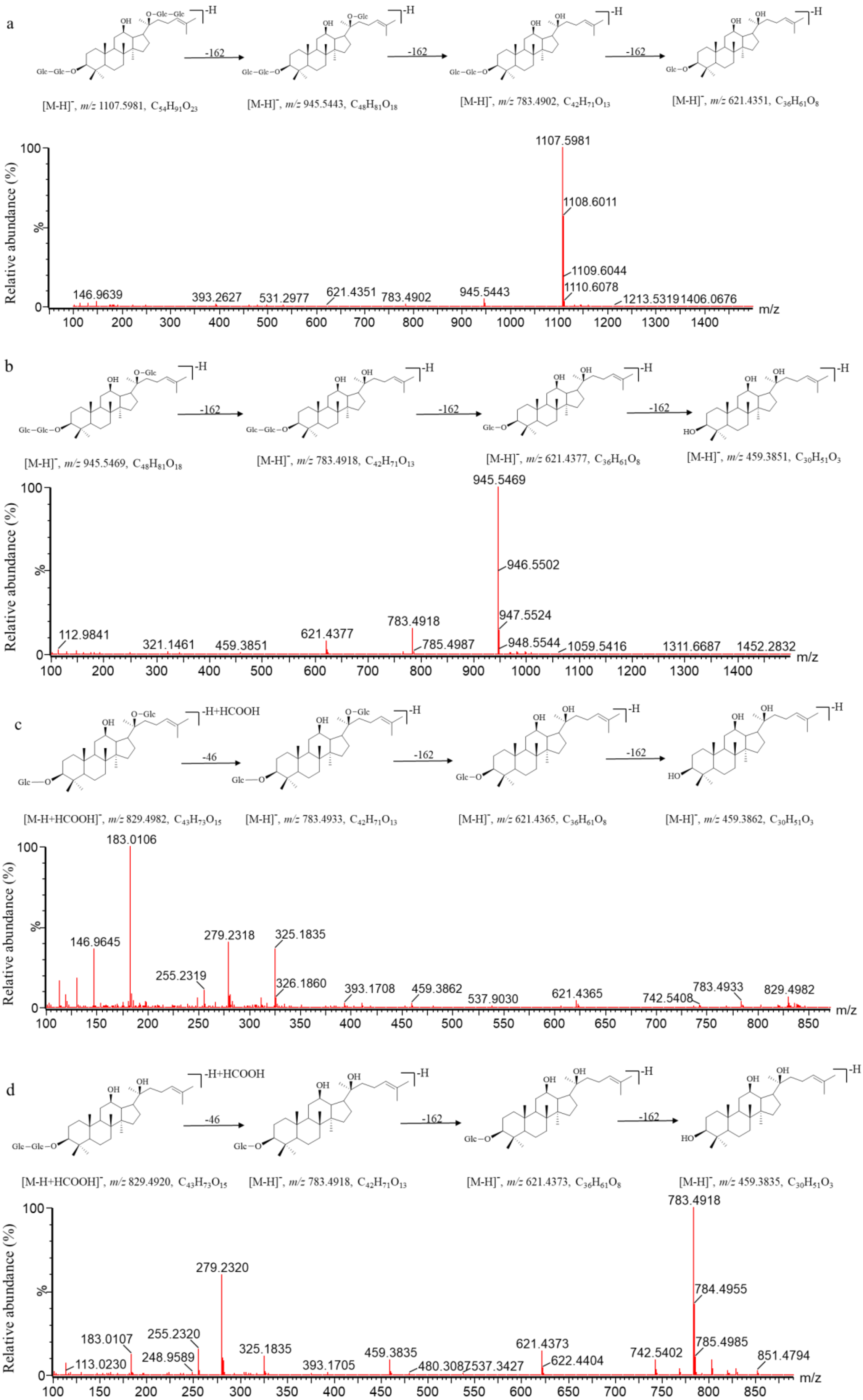
a
[M-H]-, m/z 1107.5981, C54H91O23
[M-H]-, m/z 945.5443, C48H81O18
[M-H]-, m/z 783.4902, C42H71O13
[M-H]-, m/z 621.4351, C36H61O8
-162
Relative abundance (%)
1107.5981
1108.6011
1109.6044
1110.6078
146.9639
393.2627
531.2977
621.4351
783.4902
945.5443
1213.5319
1406.0676
m/z
b
[M-H]-, m/z 945.5469, C48H81O18
[M-H]-, m/z 783.4918, C42H71O13
[M-H]-, m/z 621.4377, C36H61O8
[M-H]-, m/z 459.3851, C30H51O3
945.5469
946.5502
947.5524
948.5544
112.9841
321.1461
459.3851
621.4377
783.4918
785.4987
1059.5416
1311.6687
1452.2832
c
[M-H+HCOOH]-, m/z 829.4982, C43H73O15
[M-H]-, m/z 783.4933, C42H71O13
[M-H]-, m/z 621.4365, C36H61O8
[M-H]-, m/z 459.3862, C30H51O3
-46
183.0106
146.9645
255.2319
279.2318
325.1835
326.1860
393.1708
459.3862
537.9030
621.4365
742.5408
783.4933
829.4982
d
[M-H+HCOOH]-, m/z 829.4920, C43H73O15
[M-H]-, m/z 783.4918, C42H71O13
[M-H]-, m/z 621.4373, C36H61O8
[M-H]-, m/z 459.3835, C30H51O3
783.4918
784.4955
785.4985
279.2320
183.0107
113.0230
255.2320
248.9589
325.1835
393.1705
459.3835
480.3087
537.3427
621.4373
622.4404
742.5402
851.4794

e

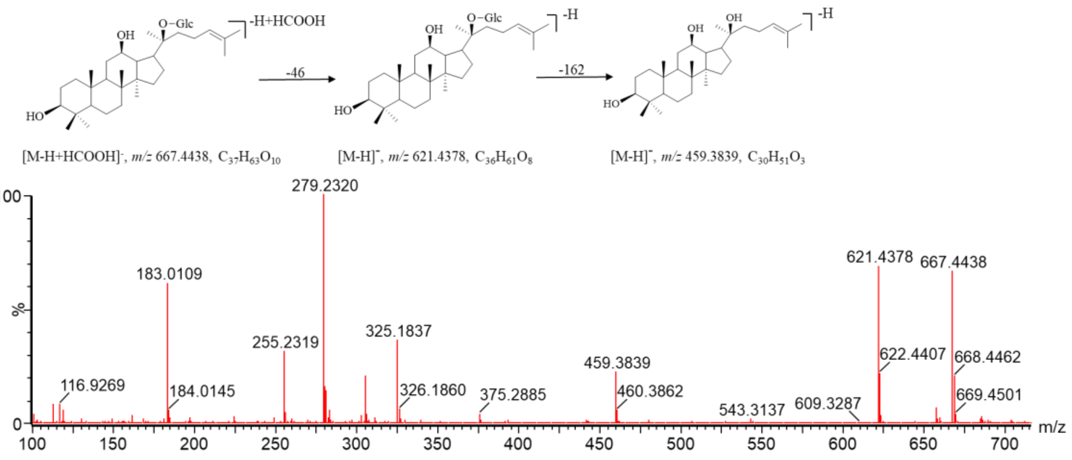

f

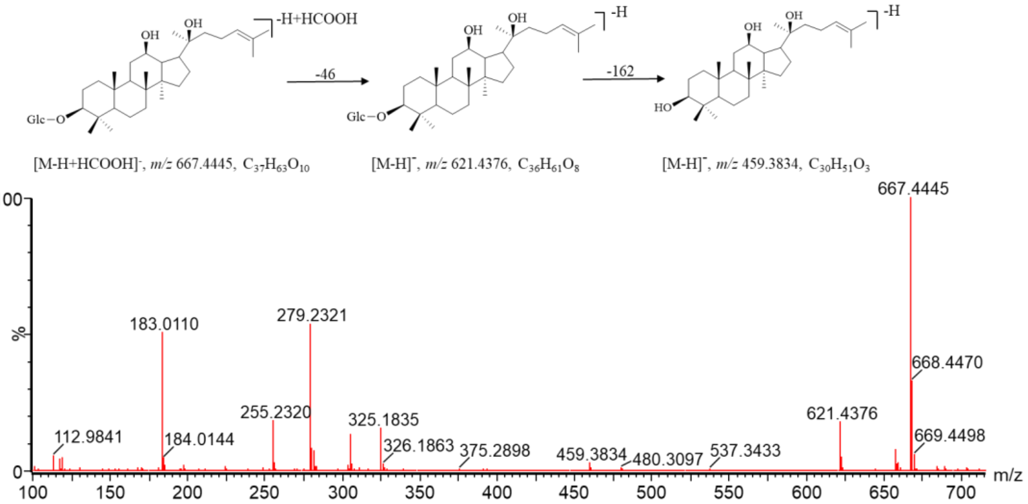

Supplementary Figure 1. MS/MS spectra and fragmentation pathways of ginsenoside Rb$_1$ and its main metabolites. **a** M0, Rb$_1$; **b** M1, Rd; **c** M2, Rg$_3$; **d** M3, F$_2$; **e** M4, Rh$_2$; **f** M5, CK.

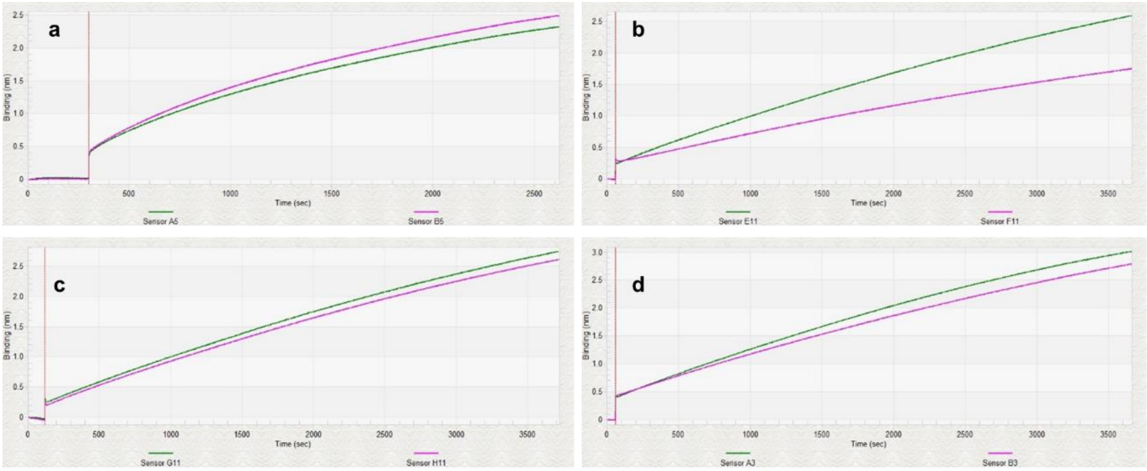

Supplementary Figure 2. Aligned loading of protein (10 μg/mL) immobilized online using Ni-NTA biosensors for the further kinetic assay with ginsenosides. a Rb_1, **b** Rd, **c** F_2, **d** Rg_3.

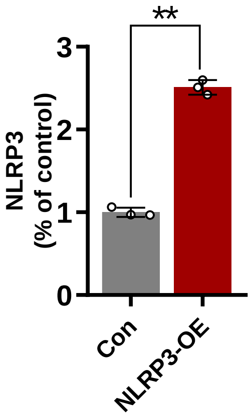

Supplementary Figure 3. Western blot analysis after macrophage transfection with NLRP3 overexpression lentiviral vectors. All data are expressed as means ± SD (n = 3). Compared with Con, $^{**}p < 0.01$.

Supplementary Table 1. Information of ginsenoside Rb_1 and its intestinal metabolites identified by UPLC-QTOF-MS/MS in negative ion mode

No.	Identification	Formula	RT	Mean measured mass (Da)	Theoretical exact mass (Da)	Mass accuracy (ppm)	Empirical formula and proposed CID fragment ions
M0	Rb_1	$C_{54}H_{92}O_{23}$	4.43	1153.6039	1153.6006	2.9	$C_{55}H_{93}O_{25}$ [M−H+HCOOH]$^-$
				1107.5981	1107.5951	2.7	$C_{54}H_{91}O_{23}$ [M−H]$^-$
				945.5443	945.5423	2.1	$C_{48}H_{81}O_{18}$ [M−H−(Glc−H_2O)]$^-$
				783.4902	783.4985	0.9	$C_{42}H_{71}O_{13}$ [M−H−2(Glc−H_2O)]$^-$
				621.4351	621.4366	-2.4	$C_{36}H_{61}O_8$ [M−H−3(Glc−H_2O)]$^-$
M1	Rd	$C_{48}H_{82}O_{18}$	5.59	991.5515	991.5478	0.6	$C_{49}H_{83}O_{20}$ [M−H+HCOOH]$^-$
				945.5469	945.5423	1.4	$C_{48}H_{81}O_{18}$ [M−H]$^-$
				783.4918	783.4895	3.7	$C_{42}H_{71}O_{13}$ [M−H−(Glc−H_2O)]$^-$
				621.4377	621.4366	2.9	$C_{36}H_{61}O_8$ [M−H−2(Glc−H_2O)]$^-$
				459.3851	459.3838	2.8	$C_{30}H_{51}O_3$ [M−H−3(Glc−H_2O)]$^-$
M2	F_2	$C_{42}H_{72}O_{13}$	11.72	829.4982	829.4949	4.0	$C_{43}H_{73}O_{15}$ [M−H+HCOOH]$^-$
				783.4933	783.4895	4.9	$C_{42}H_{71}O_{12}$ [M−H]$^-$
				621.4365	621.4366	-0.2	$C_{36}H_{61}O_8$ [M−H−(Glc−H_2O)]$^-$
				459.3862	459.3838	5.2	$C_{30}H_{51}O_3$ [M−H−2(Glc−H_2O)]$^-$
M3	Rg_3	$C_{42}H_{72}O_{13}$	11.85	829.4920	829.4949	-0.3	$C_{43}H_{73}O_{15}$ [M−H+HCOOH]$^-$
				783.4918	783.4895	2.9	$C_{42}H_{71}O_{13}$ [M−H]$^-$
				621.4373	621.4366	1.1	$C_{36}H_{61}O_8$ [M−H−(Glc−H_2O)]$^-$
				459.3835	459.3838	-0.7	$C_{30}H_{51}O_3$ [M−H−2(Glc−H_2O)]$^-$
M4	CK	$C_{36}H_{62}O_8$	12.30	667.4438	667.4421	2.5	$C_{37}H_{63}O_{10}$ [M−H+HCOOH]$^-$
				621.4378	621.4366	1.9	$C_{36}H_{61}O_8$ [M−H]$^-$
				459.3839	459.3838	0.2	$C_{30}H_{51}O_3$ [M−H−(Glc−H_2O)]$^-$
M5	Rh_2	$C_{36}H_{62}O_8$	12.37	667.4445	667.4421	3.6	$C_{37}H_{63}O_{10}$ [M−H+HCOOH]$^-$
				621.4376	621.4366	1.6	$C_{36}H_{61}O_8$ [M−H]$^-$
				459.3834	459.3838	-0.9	$C_{30}H_{51}O_3$ [M−H−(Glc−H_2O)]$^-$

Note: RT, the retention time of ginsenoside Rb1 and its metabolites. Glc, β-D-glucopyranosyl.

Supplementary Table 2. Target information related to immunomodulatory activity of ginsenosides Rb_1, Rd, Rg_3 and F_2

Number	Protein name	Gene name	Uniprot ID	Species
1	NACHT, LRR and PYD domains-containing protein 3	NLRP3	Q8R4B8	Mus musculus
2	Signal transducer and activator of transcription 3	Stat3	P42227	Mus musculus
3	RAC-alpha serine/threonine-protein kinase	AKT1	P31750	Mus musculus
4	Androgen Receptor	AR	P19091	Mus musculus
5	Caspase-3	CASP3	P70677	Mus musculus
6	Caspase-8	CASP8	O89110	Mus musculus
7	Caspase-9	CASP9	Q8C3Q9	Mus musculus
8	Cyclin-dependent kinase 1 (by homology)	CDK1	P11440	Mus musculus
9	Fibroblast growth factor 1	FGF1	P61148	Mus musculus
10	Fibroblast growth factor 2	FGF2	P15655	Mus musculus
11	Proheparin-binding EGF-like growth factor	HBEGF	Q06186	Mus musculus
12	Histone deacetylase 6	HDAC6	Q9Z2V5	Mus musculus
13	Histone deacetylase 1	HDAC1	O09106	Mus musculus
14	Histone deacetylase 8	HDAC8	Q8VH37	Mus musculus
15	Heat shock protein HSP 90-alpha (by homology)	HSP90AA1	P07901	Mus musculus
16	Interleukin-2	IL-2	Q08867	Mus musculus
17	Interleukin-6	IL-6	P22272	Mus musculus
18	Interleukin-1β	IL-1β	P10749	Mus musculus
19	Lysophosphatidic acid receptor 1	LPAR1	P61793	Mus musculus
20	Mitogen-activated protein kinase 1	MAPK1	P63085	Mus musculus
21	Mitogen-activated protein kinase 3	MAPK3	Q63844	Mus musculus
22	Matrix metalloproteinase 9	MMP9	P52176	Mus musculus
23	Nitric oxide synthase, inducible	NOS2	P29477	Mus musculus
24	BDNF/NT-3 growth factors receptor	NTRK2	P15209	Mus musculus
25	Platelet-activating factor receptor	PTAFR	Q62035	Mus musculus
26	Nuclear receptor ROR-gamma	RORC	P51450	Mus musculus
27	Tumor necrosis factor (TNF)	TNF	P06804	Mus musculus
28	NAD-dependent deacetylase sirtuin-1	SIRT1	Q923E4	Mus musculus
29	Nuclear factor NF-κB (NF-κB)	NF-κB1	P25799	Mus musculus

Supplementary Table 3. Topological features of the compound-target-pathway network

Parameters	Numerical value	Parameters	Numerical value
Clustering coefficient	0	Number of nodes	42
Connected components	1	Network density	0.141
Network diameter	4	Network heterogeneity	0.717
Network radius	3	Isolated nodes	0
Network centralization	0.351	Number of self-loops	0
Shortest paths	1640(100%)	Multi-edge node pairs	0
Characteristic path length	2.337	Analysis time (sec)	0.017
Avg. number of neighbors	5.659		

Supplementary Table 4. Topological features of the crucial shared targets

Number	Target name	Degree	Closeness Centrality	Betweenness Centrality
1	NLRP3	11	0.10468151	0.56338028
2	AKT1	10	0.08971125	0.53834951
3	MAPK1	9	0.04140534	0.44943820
4	TNF	8	0.07166097	0.51948052
5	HSP90AA1	6	0.01743861	0.42105263
6	MMP9	6	0.03105904	0.49382716
7	MAPK3	6	0.01676439	0.42105263

Supplementary Table 5. Docked interaction analysis between ginsenosides Rb_1, Rd, Rg_3, F_2 with NLRP3

Ligand name	Docking score(kcal/mol)	Number of H-bonds	Active site residues	Number of Interacting bonds
Rb_1	-9.28	1	THR167	1
Rd	-10.95	3	ARG168	1
			ARG165	1
			LYS375	1
Rg_3	-12.33	2	PHE408	1
			TYR441	1
F_2	-13.64	1	TYR379	1

Supplementary Table 6. Kinetic parameters of interaction between ginsenosides Rb_1, Rd, Rg_3, F_2 with NLRP3

Compounds	K_D (M)	K_D error	k_{on}(1/Ms)	k_{on} error	k_{dis}(1/s)	k_{dis} error	Full R^2
Rb_1	9.96E-05	1.19E-05	1.34E+03	1.17E+02	1.34E-01	1.09E-02	0.8567
Rd	5.01E-05	2.93E-06	1.48E+03	6.59E+01	7.39E-02	2.78E-03	0.9061
Rg_3	9.61E-06	1.75E-06	1.77E+04	2.39E+03	1.70E-01	2.07E-02	0.8388
F_2	6.16E-05	8.01E-06	3.61E+03	3.45E+02	3.45E+02	1.96E-02	0.8615

Supplementary Table 7. Mass spectrometry conditions for pharmacokinetic analysis

Compounds	Ion channels	Ion mode	Ion transition (*m/z*→*m/z*)	Taper hole voltage (V)	Cone colliding energy (V)	Retention time (min)	Time period (min)
Digoxin	I	ESI	803.7→283.5	70	50	4.02	0-7
Rb_1			1131.9→365.3	80	60	4.73	
Rd			970.0→789.6	70	50	6.09	
F_2	II		807.8→203.2	70	40	8.36	7-15.5
Rg_3			808.0→365.2	85	50	9.35	
Rh_2			621.3→161.1	80	50	12.10	
CK			645.7→203.2	70	40	12.25	

Supplementary Table 8. The primer sequences for RT-qPCR

Gene	Forward	Reverse
iNOS	CATGCTACTGGAGGTGGGTG	CATTGATCTCCGTGACAGCC
IL-1β	AGGACATGAGCACCTTCTTTTC	ACGTCACACACCAGCAGGTTA
TNF-α	TGCCTATGTCTCAGCCTCTTC	GAGGCCATTTGGGAACTTCT
IL-4	GTTCTTCGTTGCTGTGAGGAC	TGTACCAGGAGCCATATCCAC
IL-10	CAGTCGGCCAGAGCCACAT	CTTGGCAACCCAAGTAACCCT
β-actin	AGTGTGACGTTGACATCCGT	GCAGCTCAGTAACAGTCCGC

Supplementary Table 9. Measurement parameter for kinetic assay using biolayer interferometry

Compounds	Step	Step name	Time(s)	Shake speed(rpm)	Step type
Rb_1	1	Baseline1	60	1000	Equilibration
	2	Loading	3600	1000	Protein loading
	3	Baseline2	60	1000	Baseline
	4	Association	600	1000	Association
	5	Dissociation	600	1000	Dissociation
Rd	1	Baseline1	60	1000	Equilibration
	2	Loading	3600	1000	Protein loading
	3	Baseline2	60	1000	Baseline
	4	Association	300	1000	Association
	5	Dissociation	300	1000	Dissociation
Rg_3	1	Baseline1	60	1000	Equilibration
	2	Loading	3600	1000	Protein loading
	3	Baseline2	60	1000	Baseline
	4	Association	240	1000	Association
	5	Dissociation	240	1000	Dissociation
F_2	1	Baseline1	60	1000	Equilibration
	2	Loading	3600	1000	Protein loading
	3	Baseline2	60	1000	Baseline
	4	Association	180	1000	Association
	5	Dissociation	180	1000	Dissociation
