# Supplementary material for: Structural analogues in herbal medicine ginseng hit a shared target to achieve cumulative bioactivity
Source: Commun Biol. 2021 May 10;4:549. doi: 10.1038/s42003-021-02084-3 (PMC8110997; doi:10.1038/s42003-021-02084-3)
Supplement: Supplementary file 2 — Description of Additional Supplementary Files [file 42003_2021_2084_MOESM2_ESM.pdf]

Description of Additional Supplementary Files

File Name: Supplementary Data 1

Description: Source data file for all graphs presented in the paper.
