# Supplementary material for: Structural analogues in herbal medicine ginseng hit a shared target to achieve cumulative bioactivity
Source: Commun Biol. 2021 May 10;4:549. doi: 10.1038/s42003-021-02084-3 (PMC8110997; doi:10.1038/s42003-021-02084-3)
Supplement: Supplementary file 4 — Reporting summary [file 42003_2021_2084_MOESM4_ESM.pdf]

nature research

Corresponding author(s): DBPR COMMSBIO-20-3674A

Last updated by author(s): Mar 22, 2021

Reporting Summary

Nature Research wishes to improve the reproducibility of the work that we publish. This form provides structure for consistency and transparency in reporting. For further information on Nature Research policies, see our Editorial Policies and the Editorial Policy Checklist.

Statistics

For all statistical analyses, confirm that the following items are present in the figure legend, table legend, main text, or Methods section.

n/a	Confirmed	
☐	☒	The exact sample size (n) for each experimental group/condition, given as a discrete number and unit of measurement
☐	☒	A statement on whether measurements were taken from distinct samples or whether the same sample was measured repeatedly
☐	☒	The statistical test(s) used AND whether they are one- or two-sided<br>*Only common tests should be described solely by name; describe more complex techniques in the Methods section.*
☒	☐	A description of all covariates tested
☒	☐	A description of any assumptions or corrections, such as tests of normality and adjustment for multiple comparisons
☐	☒	A full description of the statistical parameters including central tendency (e.g. means) or other basic estimates (e.g. regression coefficient) AND variation (e.g. standard deviation) or associated estimates of uncertainty (e.g. confidence intervals)
☐	☒	For null hypothesis testing, the test statistic (e.g. F, t, r) with confidence intervals, effect sizes, degrees of freedom and P value noted<br>*Give P values as exact values whenever suitable.*
☒	☐	For Bayesian analysis, information on the choice of priors and Markov chain Monte Carlo settings
☒	☐	For hierarchical and complex designs, identification of the appropriate level for tests and full reporting of outcomes
☒	☐	Estimates of effect sizes (e.g. Cohen's d, Pearson's r), indicating how they were calculated

Our web collection on statistics for biologists contains articles on many of the points above.

Software and code

Policy information about availability of computer code

Data collection	MassLynxTM (v 4.1) software was used for serum pharmacochemistry and pharmacokinetics data collection; Octet Data Analysis (v7.0) software was used for biolayer interferometry assay data collection.
Data analysis	MassLynxTM (v 4.1) software was used for serum pharmacochemistry data analysis; NIH Image J (v1.52) software was used for western blot and immunofluorescence microscopy data analysis; AutoDock (v4.2.6) software was used for molecular docking data analysis; Octet Data Analysis (v7.0) software was used for biolayer interferometry assay data analysis; Others were analysed by GraphPad Prism (v 8.0.1).

For manuscripts utilizing custom algorithms or software that are central to the research but not yet described in published literature, software must be made available to editors and reviewers. We strongly encourage code deposition in a community repository (e.g. GitHub). See the Nature Research guidelines for submitting code & software for further information.

Data

Policy information about availability of data

All manuscripts must include a data availability statement. This statement should provide the following information, where applicable:

- Accession codes, unique identifiers, or web links for publicly available datasets
- A list of figures that have associated raw data
- A description of any restrictions on data availability

The datasets generated during and/or analyzed during the current study are available from the corresponding author on reasonable request. Source data underlying plots shown in main figures are provided in Supplementary Data.

Field-specific reporting

Please select the one below that is the best fit for your research. If you are not sure, read the appropriate sections before making your selection.

- [x] Life sciences
- [ ] Behavioural & social sciences
- [ ] Ecological, evolutionary & environmental sciences

For a reference copy of the document with all sections, see nature.com/documents/nr-reporting-summary-flat.pdf

Life sciences study design

All studies must disclose on these points even when the disclosure is negative.

Sample size	Ten samples in parallel for in vivo assay and three samples in parallel for in vitro assay.
Data exclusions	No data were excluded from the analyses.
Replication	In pharmacokinetics assay, intra- and inter-day variations were chosen to determine the precision of the developed HPLC-TQ-MS analysis. For intra-day variability test, the serum sample was extracted and analyzed for six replicates within one day, while for inter-day variability test, the same sample was examined in duplicates for three consecutive days.
Randomization	The mice were randomly divided into 5 groups (10 for each group): control group (Con), model group (Mod) and three Rb1-treated groups, namely the low-dose (L, 40 mg/kg), middle-dose (M, 80 mg/kg) and high-dose (H, 160 mg/kg) groups.
Blinding	Blind experiment was not involved in our study .

Reporting for specific materials, systems and methods

We require information from authors about some types of materials, experimental systems and methods used in many studies. Here, indicate whether each material, system or method listed is relevant to your study. If you are not sure if a list item applies to your research, read the appropriate section before selecting a response.

Materials & experimental systems

n/a	Involved in the study	
☐	☒	Antibodies
☐	☒	Eukaryotic cell lines
☒	☐	Palaeontology and archaeology
☐	☒	Animals and other organisms
☒	☐	Human research participants
☒	☐	Clinical data
☐	☒	Dual use research of concern

Methods

n/a	Involved in the study	
☒	☐	ChIP-seq
☐	☒	Flow cytometry
☒	☐	MRI-based neuroimaging

Antibodies

Antibodies used	For Western blot: the primary antibodies including anti-NLRP3 (ab214185), anti-Interleukine-18 (IL-18, ab71495) and anti-NEK7 (ab133514) were purchased from Abcam Co. (Cambridge, UK); anti-Caspase-1(#24232), cleaved anti-Caspase-1(#89332), anti-IL-1β (#12242), cleaved anti-IL-1β (#52718), anti-ASC (#67824) and anti-GAPDH (#5174) were purchased from Cell Signaling Technology (Danvers, MA, USA); the second antibody Goat Anti-Rabbit IgG H&L (ab6702) was purchased from Abcam Co. (Cambridge, UK).<br>For Immunofluorescence microscopy: NLRP3 antibody (ab270449) and Goat Anti-Rabbit IgG H&L (Alexa Fluor 594) antibody (ab150080) were purchased from Abcam Co. (Cambridge, UK).
Validation	Species and application of all antibodies were validated.

Eukaryotic cell lines

Policy information about cell lines

Cell line source(s)	Mouse RAW264.7 cells (Cell Bank of the Chinese Academy of Sciences, Shanghai, China)
Authentication	SIg-, Ia-antigen, Thy-1.2 surface antigen is negative. This cell line does not secrete detectable virus particles, and the XC spot formation test is negative. LPS or PPD treatment for 2 days can induce the decomposition of red blood cells but has no effect on tumor target cells. Passed mycoplasma detection in National Collection of Authenticated Cell Cultures.
Mycoplasma contamination	All cell lines negative for mycoplasma contamination.

Commonly misidentified lines (See ICLAC register)	No misidentified cell lines were found.

Animals and other organisms

Policy information about studies involving animals; ARRIVE guidelines recommended for reporting animal research

Laboratory animals	Male ICR mice, 6-8 weeks of age
Wild animals	Wild animals were not involved in our study.
Field-collected samples	Each animal was evaluated to be in good health, and then acclimated to the laboratory environment (12 h light/dark cycle, at 23-27℃, and 30-60% relative humidity) for one week before experiments. Feed and potable water were provided ad libitum.
Ethics oversight	Animal facilities and protocols were approved by the Animal Ethics Committee of Jiangsu Province Academy of Traditional Chinese Medicine. All procedures were conducted in strict accordance with Guide for the Care and Use of Laboratory Animals of the National Institutes of Health (NIH Publication No. 80-23; revised in 1978).

Note that full information on the approval of the study protocol must also be provided in the manuscript.

Dual use research of concern

Policy information about dual use research of concern

Hazards

Could the accidental, deliberate or reckless misuse of agents or technologies generated in the work, or the application of information presented in the manuscript, pose a threat to:

No	Yes	
☒	☐	Public health
☒	☐	National security
☒	☐	Crops and/or livestock
☒	☐	Ecosystems
☒	☐	Any other significant area

Experiments of concern

Does the work involve any of these experiments of concern:

No	Yes	
☒	☐	Demonstrate how to render a vaccine ineffective
☒	☐	Confer resistance to therapeutically useful antibiotics or antiviral agents
☒	☐	Enhance the virulence of a pathogen or render a nonpathogen virulent
☒	☐	Increase transmissibility of a pathogen
☒	☐	Alter the host range of a pathogen
☒	☐	Enable evasion of diagnostic/detection modalities
☒	☐	Enable the weaponization of a biological agent or toxin
☒	☐	Any other potentially harmful combination of experiments and agents

Flow Cytometry

Plots

Confirm that:

☒ The axis labels state the marker and fluorochrome used (e.g. CD4-FITC).

☒ The axis scales are clearly visible. Include numbers along axes only for bottom left plot of group (a 'group' is an analysis of identical markers).

☐ All plots are contour plots with outliers or pseudocolor plots.

☒ A numerical value for number of cells or percentage (with statistics) is provided.

Methodology

Sample preparation	Raw 264.7 cells in serum-free DMEM were added to a 24-well inner transwell chamber (8 μm, Corning Inc.), which was pre-coated with or without 300 μg/mL Matrigel (BD Biosciences) for 2 h. The outer chamber was filled with 10% FBS in DMEM

	medium containing the indicated concentration of ginsenosides (individuals or combination) as an inducer. Cells were stimulated by 1 μg/mL LPS (dissolved in DMEM) for 24 h, and 5 mM ATP was added for another 30 min. After discarding the medium, 0.05% trypsin (without EDTA) was added for digestion. Cell precipitates were collected, and washed twice with PBS for propidium iodide (Beyotime, C1052) staining, and the cell pyroptosis rate was detected.
Instrument	FACS CantoII flow cytometry (Becton Dickinson, CA)
Software	CytExpert
Cell population abundance	5×10^4 cells/well
Gating strategy	This strategy was not used in the detection of cell pyroptosis.

☐ Tick this box to confirm that a figure exemplifying the gating strategy is provided in the Supplementary Information.
